# Does exercise influence burn-induced inflammation: A cross-over randomised controlled feasibility trial

**DOI:** 10.1371/journal.pone.0266400

**Published:** 2022-04-01

**Authors:** Grant Rowe, Dale W. Edgar, Tyler Osborne, Mark Fear, Fiona M. Wood, Pippa Kenworthy

**Affiliations:** 1 Fiona Wood Foundation, Fiona Stanley Hospital, Perth, Western Australia, Australia; 2 Burns Service of Western Australia, Fiona Stanley Hospital, South Metropolitan Health Service, Perth, Western Australia, Australia; 3 Institute for Health Research, The University of Notre Dame Australia, Fremantle, Western Australia, Australia; 4 Burn Injury Research Unit, University of Western Australia, Crawley, Australia; 5 Disciple of Exercise Physiology, Murdoch University, Murdoch, Australia; Prince Sattam Bin Abdulaziz University, College of Applied Medical Sciences, SAUDI ARABIA

## Abstract

**Background:**

Burn injuries trigger a greater and more persistent inflammatory response than other trauma cases. Exercise has been shown to positively influence inflammation in healthy and diseased populations, however little is known about the latent effect of exercise on chronic inflammation in burn injured patients. The aims of the pilot study were to assess the feasibility of implementing a long duration exercise training program, in burn injured individuals including learnings associated with conducting a clinical trial in COVID-19 pandemic.

**Methods:**

Fifteen participants with a burn injury between 5–20% total body surface area acquired greater than a year ago were randomised in a within-subject designed study, into one of two conditions, exercise–control or control–exercise. The exercise condition consisted of six weeks of resistance and cardiovascular exercises, completed remotely or supervised in a hospital gym. A comprehensive outcome measurement was completed at the initial, mid and end point of each exercise and control condition. To determine the success of implementation, the feasibility indicator for the data completeness across the comprehensive outcome battery was set at 80%.

**Results:**

Half (49%) of eligible participants in the timeframe, were recruited and commenced the study. Six participants withdrew prior to completion and a total of 15 participants completed the study. Eight participants were randomised to the exercise-control and seven to the control exercise group. Five participants trained remotely and seven did supervised training. Three participants completed a mix of both supervised and remote training initiated due to COVID restrictions. Outcome measures were completed on 97% of protocolised occasions and 100% of participants completed the exercise training.

**Conclusions:**

Conducting a long duration exercise training study on burn injured individuals is feasible using the described methods. The knowledge gained helps improve the methodology in larger-scale projects. Insights into the impact of COVID-19 on this clinical trial and success enhancing adaptations for the researcher, research practice and the participant, are presented.

## Introduction

Burn injury is a significant global public health problem; with an estimated 11 million incidences occurring per year, accounting for over 300,000 deaths [1]. Contemporary research focus is shifting to better understanding the links between acute traumatic effects and surgical interventions and biopsychosocial and, or scarring outcomes, as non-fatal burn injury is a leading cause of long-term morbidity [2, 3]. The non-fatal burden of disease, estimated using the INTEGRIS method, is 281 and 279 years lost to disability (YLD) per 100,000 inhabitants for Australia and New Zealand respectively [2]. Sufferers are more likely to develop secondary pathologies caused by chronic persistent homeostatic disruptions to their endocrine and immune systems than the uninjured community [4]. Although the expression of stress hormones is a natural response to trauma, their prolonged presence can lead to immunosuppression [5], increasing the risk of developing cardiovascular disease, diabetes, and cancer [6, 7].

Despite the acceptance of the latent impact of burn injuries, there is little research that has investigated interventions to mitigate these confirmed sequelae. An affordable and accessible intervention that has been shown to positively influence these outcomes in healthy and diseased populations is regular exercise [8, 9]. Although those unaccustomed to exercise can exhibit acutely increase pro-inflammatory cytokines, such as interlukin-6 (IL-6) [10], regular exercise can decrease resting systemic levels of pro-inflammatory cytokines as well as drive the cytokine balance into an anti-inflammatory state [8, 11, 12]. As a burn injury triggers a greater and more persistent inflammatory response than any other trauma [13], investigating the influence of exercise on burn-induced inflammation may offer a novel and cost effective approach to mitigate stress responses in burn injury sufferers.

However, there is an absence of well-designed randomised controlled trials (RCT) that have implemented structured exercise in those who have previously suffered a burn injury. Early evidence indicates that strength training undertaken by burn injury inpatients in the acute recovery phase can limit resting systemic inflammation [14]. However, little is known about the training effects on inflammation generated in individuals with a non-severe burn injury beyond the acute wound recovery period. The median length of stay in hospital for an adult suffering a burn injury in Western Australia is 3 days [15], with many patients no longer requiring treatment or follow up a year after injury. Researchers and clinicians therefore need to be aware of the challenges of conducting exercise training and designing studies in this cohort of individuals. The primary aim of this feasibility trial ‘Exercise and Burn-induced Inflammation’ (ExBIN) was to assess the methods of conducting an exercise training study in individuals with old burn injuries including, participant recruitment and study commitment adherence. The secondary aim was to explore the learnings associated with conducting a clinical trial during the COVID-19 pandemic.

### Methods

The detailed methodology presented below describes the clinical trial protocol (S1 File) assessed during this feasibility study. The detail provides information necessary to reproduce the study protocol in other burn services and understand the complexity of conducting long duration exercise intervention studies and commitment necessary by participants for study success.

### Trial design

A within-subject RCT, with participants randomised to either the exercise–control (EX-CON) cross-over or the control–exercise (CON-EX) cross-over was conducted between September 2020 and July 2021. The study was approved by the South Metropolitan Health Service Human Research Ethics Committee (RGS3381) and registered on the Australian and New Zealand Clinical Trials Registry (ACTRN12620000237987p).

### Participants

Participants were recruited if they had sustained a 5–20% total body surface area (TBSA) burn injury over one (1) year ago, were >18 years old and were able to provide written consent. They also had to have satisfactory self-described health status (excluding burn injury) and live within the Greater Perth metropolitan region, Western Australia. They were excluded if they had: an acquired or pre-existing neurological injury or disease conditions which influenced the capacity to complete an exercise or walking program, e.g., spinal cord injury, stroke, and central nervous system lesions; unstable cardiac conditions; were intellectually challenged; non-English speaking; pregnant; cancer; were not willing to stop deliberate strength training during study period; were rural and remote West Australian residents; scheduled to undergo reconstructive surgery or receive laser during the study period.

Eligible participants were identified from retrospective electronic patient records and recruited when they attended their routine multidisciplinary team hospital appointment or contacted via telephone.

**Fig 1:**
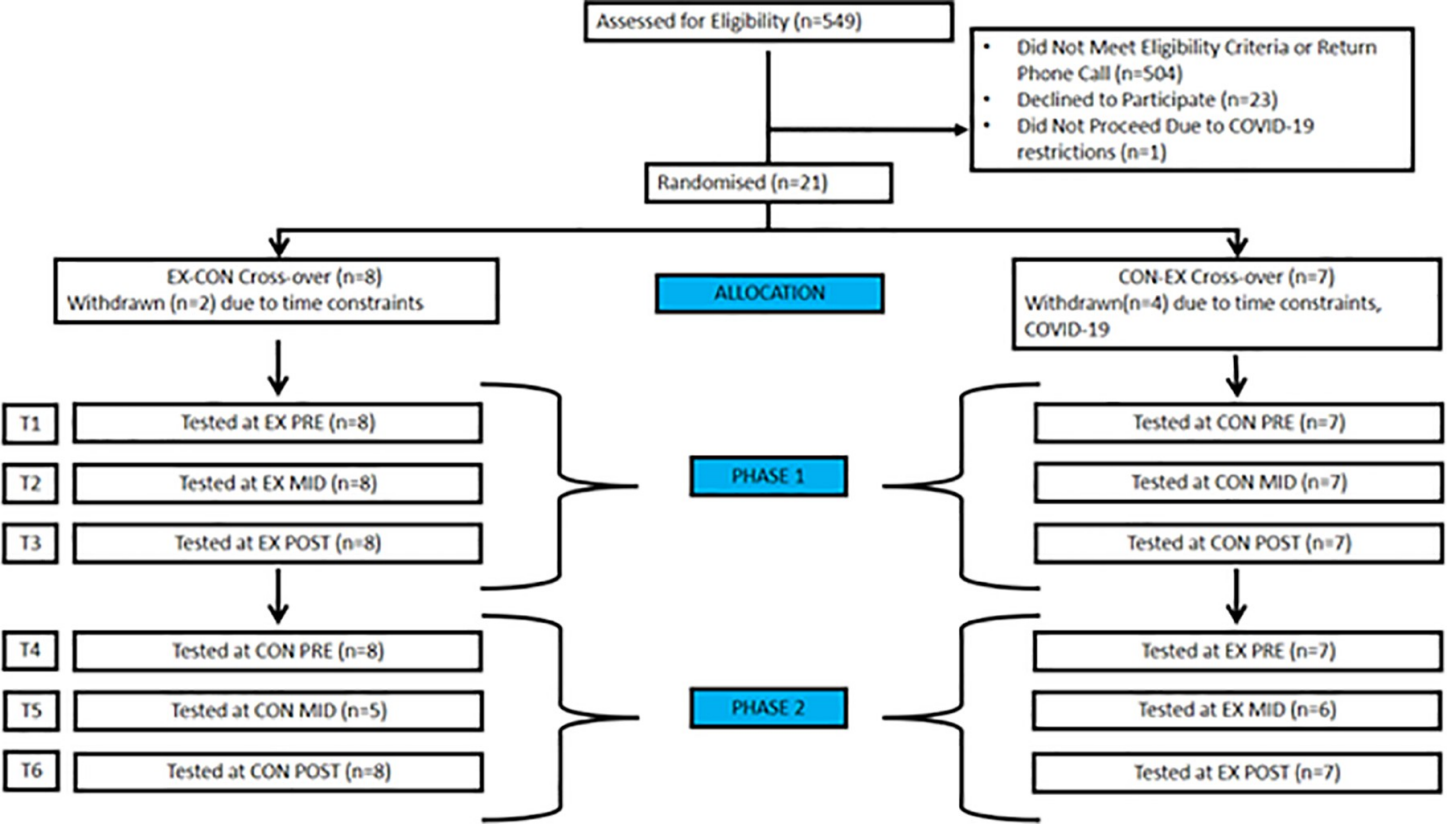
Flow of participants through study. T1: time point 1 (initial of phase 1); T2: time point 2 (mid way of phase 1); T3: time point 3 (final of phase 1); T4: time point 4 (initial of phase 2); T5: time point 5 (mid way of phase 2); T3: time point 3 (final of phase 1); T6: time point 6 (final of phase 2); EX-CON: exercise–control condition; CON-EX: control–exercise condition.

After providing consent participants were assigned into the EX-CON or the CON-EX group. Allocation to the groups was achieved using a random generation formula in Microsoft Excel. Participation in the study was for 16 weeks (Fig 1) and participants attended a total of six outcome assessment sessions.

### Exercise condition

Participants were presented with two exercise intervention options: 1) supervised training at the hospital, or 2) remote training on their own, with exercise support and monitoring using a smartphone app called PhysiApp® (Physitrack Limited, London, UK). All participants who undertook remote training were deemed ‘low risk of adverse events to exercise’ on the Patient Health Questionnaire (PHQ) (South Metropolitan Health Service, Fiona Stanley Fremantle Hospitals Group). For both exercise options, the exercises were prescribed and/or supervised by an accredited Exercise and Sports Scientist, Exercise Physiologist, or Physiotherapist. The exercise intervention was 6-weeks long, with the participants expected to complete three training sessions per week and walk, with moderate intensity for 20-minutes on the non-training days. The training sessions aimed to achieve 60-minutes of moderate-to-vigorous activity consisting of both strength and cardiorespiratory training.

Participants who undertook supervised training were prescribed six multi-joint strength exercises consisting of squats, leg press, hip bridges, chest press, shoulder press and seated row variations. All strength exercises were completed to near or complete failure against a load that enabled the participants to complete 8–12 repetitions for each set. Previous reports indicate that performing near fatiguing sets of 10 repetitions is equivalent to approximately lifting a 70% one repetition-maximum (1-RM) load to near failure [16]. Three sets were completed for each exercise, with the participants alternating between upper- and lower-body exercises to ensure sessions were completed timely. Cardiorespiratory training consisted of completing a session total of 20-min of stationary cycling at a rating of perceived exertion (RPE) of 6–7 [17], preferentially completed as two 10 minute blocks of activity dispersed between the strength exercises. Participants who undertook the remote training were prescribed a similar strength and cardiorespiratory training program, with the same instruction to complete the 8–12 repetition strength sets to near failure. However, due to equipment access discrepancies, there were variations to the exercises and the number of repetitions performed during sets, especially for cardiorespiratory training, where participants performed either running, cycling and body weight movements such as star jumps and mountain climbers (plank position with alternating pulling knees to chest). Home-based strength equipment consisted of a strong bungee and resistance bands if access to weights were not available. Although these bands provide strong resistance when stretched, it was noted that some participants required more repetitions to reach near failure for strength exercises.

Participants could continue participating in leisure or recreational sporting activities throughout the study.

### Control condition

For the control period, participants were advised to maintain or return to their normal physical activity levels (except if applicable, participants were asked to refrain from strength training).

### Wash out period

Participants were instructed to cease all exercise training but could continue with light activity such as light walking.

### Outcome measurement

**Fig 2:**
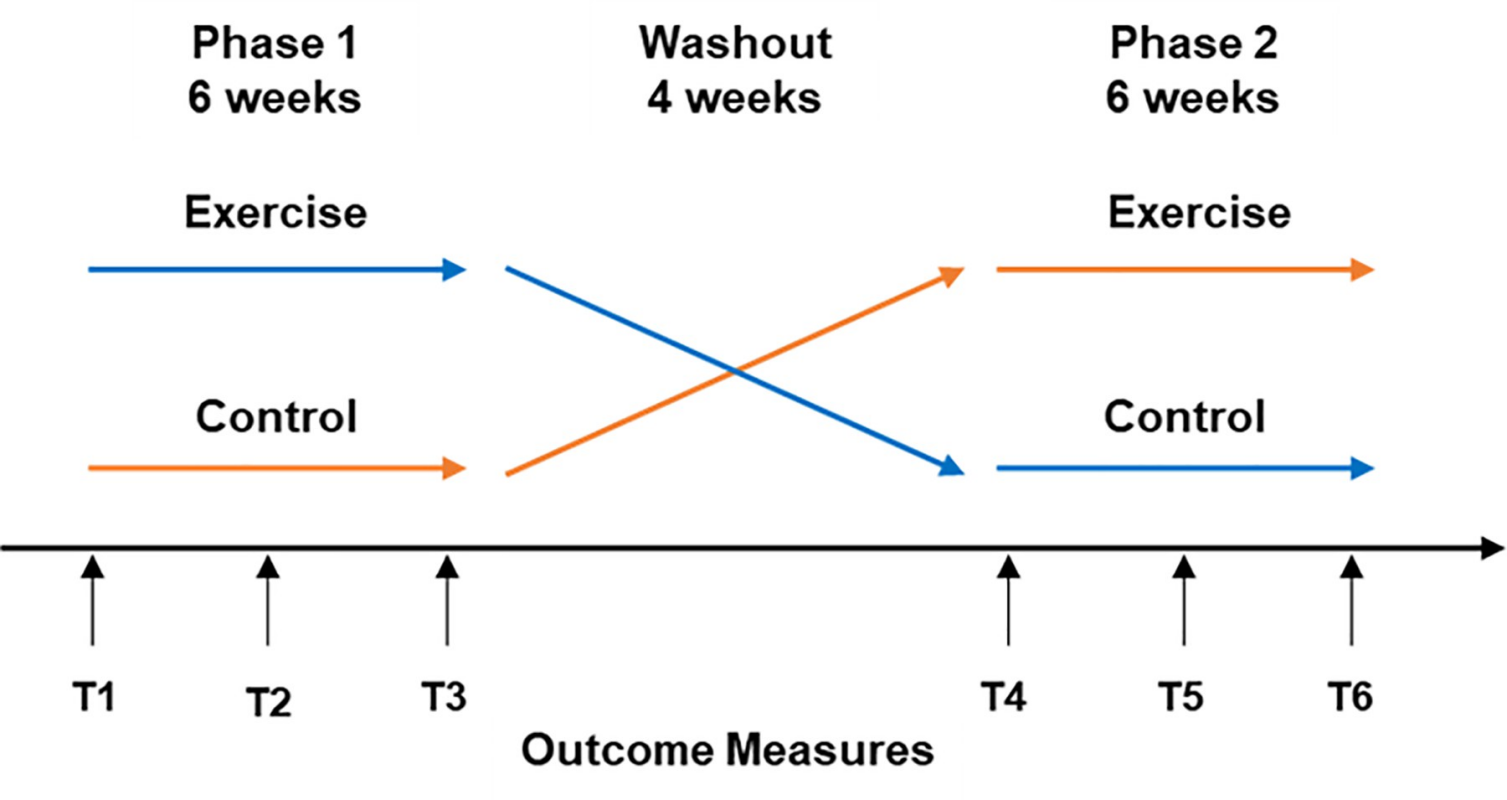
Experimental protocol of the study. T1: time point 1 (initial of phase 1); T2: time point 2 (mid way of phase 1); T3: time point 3 (final of phase 1); T4: time point 4 (initial of phase 2); T5: time point 5 (mid way of phase 2); T6: time point 6 (final of phase 2).

Participants attended a minimum of six testing sessions throughout the study period. Assessments were completed at these times including a baseline at the beginning (T1, T4), mid-point testing (T2, T5) and a final battery at the end (T3, T6) of each phase (Fig 2). A four week wash-out was prescribed between the phases to limit training residual effects on inflammatory markers affecting the control period results when participants performed the EX-CON cross-over [14].

#### Primary outcome

As a feasibility trial, the primary outcome was to assess the study methodology options in relation to outpatient recruitment and study commitment adherence in participants who undertook either supervised training at the hospital or remote training with support of a smartphone app.

#### Physiological and physical outcomes

Clinical outcomes were collected as per the protocol above and to determine the success of implementation, the feasibility indicator for the data completeness across the comprehensive outcome battery was set at 80%. A feasibility indicator for participant adherence to training (training sessions complete) was also set at 80%. The outcome measures for the clinical trial included inflammatory biomarker TNF alpha, physiological (non-invasive muscle mass bioimpedance spectroscopy [18, 19]); muscle strength (isometric grip and bicep and quad strength [20]); cardiorespiratory fitness—modified Chester Step Test [21]; Self-reported physical functional (QuickDASH–upper limb function [22], LLFI- lower limb function [23]); neurological (Semmes-Weistein monofilaments, two-point discrimination, proprioception) [24]; metabolome analysis (urine and hair samples, plasma (multiplex of other cytokines)); resting metabolic rate and metabolic flexibility using indirect calorimetry (COSMED Quark-RMR, Rome, Italy); quality of life (SF36 [25]) and self-reported activity survey (International Physical Activity Questionnaire (IPAQ)).

### Statistical analysis

Descriptive statistics were used to describe participant characteristics, study commitment adherence and the feasibility of the study. Variables were described as medians and interquartile range (IQR).

## Results

During the study recruitment period (30 September 2020–31 March 2021), 549 outpatients meeting the inclusion criteria were screened for eligibility to participate in the study. Five hundred and four (n = 504) of these outpatients did not meet the inclusion criteria (predominantly due to living outside the Greater Perth region), could not be contacted due to outdated details, or did not return phone calls. The flow of participants through the study is demonstrated in Fig 1. Twenty-two (22) of the 45 (49%) outpatients contacted consented to participate. One outpatient was unable to commence due to COVID-19 hospital restrictions and six (6) participants (28%) withdrew from the study. Three participants completed the initial assessment (T1) only, two in CON-EX group completed T1-T3 only and one in CON-EX group completed T1-T4. Fifteen (15) of 21 participants completed the final testing session. Of the participants that completed the study, eight were randomised to the EX-CON cross-over and seven were randomised to the CON-EX cross-over (Fig 1). Participant characteristics are compared in Table 1.

**Table 1:** Sample descriptive characteristics.

Participants (*n*)		
Exercise-Control Group	8	
Control-Exercise Group	7	
Total	15	
**Male/Female**	4 (27%) / 11 (73%)	
**Age (years) [median (IQR)]**	41 (28–58)	
**Total Body Surface Area (%) [median (IQR)]**	9% (6.5–12.5)	
**Location of Burn (*n*)**		
Upper Limb	2	Additional burn areas: 7 trunk 2 face 1 trunk and face.
Lower Limb	7	
Upper and lower limb	6	
**Burn Depth**		
Superficial partial thickness	85.7%	
Deep partial thickness	14.3%	
**Time after injury (years) [median (IQR)]**	2.9 (2.50–3.45)	

Outcome assessment sessions took on average 70 minutes to complete without the metabolic testing and an average of 130 minutes with metabolic testing.

Five participants performed exercise training remotely, seven had supervised training at Fiona Stanley Hospital Burns gym and three participants completed a mix of remote and supervised training. Only one participant was required to do supervised training as assessed on the PHQ. All 15 participants, irrespective of the training method, completed 100% of their exercise sessions. Additionally, 100% of participants training remotely were compliant with updating completion of their exercise sessions via the app, Physiapp®.

### Outcome measures

Data completeness results across the comprehensive outcome battery, with success set at 80%, are reported in Table 2 and the study’s minimal data set is included as (S1 Dataset).

**Table 2:** Capture success rate of outcome measures: six assessment session per participant (n = 15).

Outcome Measures		Success of outcome measure assessment
Exercise Risk Screen	Patient Health Questionnaire (PHQ)–Assessed once only at prior to exercise phase	15/15*
Inflammatory Biomarker	TNF* alpha*	90/90
Physiological and Physical	Bioimpedance Spectroscopy	87/90
	Muscle Strength	
	• Quadriceps • Biceps • Grip	89/90 89/90 90/90
Fitness/activity	Modified Chester step test	89/90
	International Physical Activity Questionnaire (IPAQ)	87/90
Functional (subjective)	Quick Dash	54/56^
	Lower limb functional Index	75/78^+^
Neurological	Semmes-Weinstein monofilaments	86/90
	Two-point discrimination	86/90
	Proprioception	90/90
Quality of Life	SF36	77/90
Metabolome analysis	Plasma (multiplex of other cytokines),	90/90
	Urine	90/90
	Hair	90/90
Metabolic testing	Calorimetry (assessed twice only per participant, pre and post exercise period)	28/30^#^

*only 1 survey completed per participant prior to exercise phase to determine eligibility for remote training

^8 participants had upper limb burns

^+^13 participants had lower limb burns

^#^Assessed only in the exercise phase at the initial assessment and the final assessment.

## Discussion

Conducting a long duration exercise training study on burn injured individuals is feasible using the described methods. Data completeness across the comprehensive outcome battery was achieved on 97% of planned occasions for those recruited surpassing the feasibility indicator of 80% (Table 2). The comprehensive outcome measurement battery took on average 70 minutes to complete and was potentially a significant burden on participants, however the high rate of adherence to outcome measurement collection and exercise intervention suggests it is achievable. In full disclosure, the original research protocol did include a microbiome measure (rectal swab) however for the feasibility study we chose not to include this as senior researchers determined it could be too much of a deterrent to participation (and would require additional funding to be sought, to analyse results). Only four of the 17 assessments required the participant to physically exert themselves, the remainder were not physically demanding and all, except one participant, completed the physical assessment measures (Table 2). The one participant did not complete the bicep and quadricep strength, in only one session, due to time constraints of the participant. Surveys were handed to participants in a bundle of four surveys to complete independently either on site or to take home and then return via email to limit the assessment time spent at the hospital. These therefore had a lower rate of completion (Table 2). Additionally, if the survey was not completed correctly, it was marked as incomplete. There were seven SF36 surveys not filled in and six which were incomplete. One participant with a lower limb burn did not return their T1 surveys even with follow up requests. One participant missed both midpoint assessments (T2 and T5) due to separate periods of COVID-19 restrictions, they had both lower limb and upper limb burns. The other surveys not completed were missed accidentally within the survey bundle. To minimise missing data, for future studies the surveys could be provided in an online format where completion of the survey is required before it can be submitted. Another and recommended option would be to request the participants complete the surveys, onsite, immediately preceding their physical assessments or at the time of the other outcome measures to ensure they are completed correctly.

One hundred percent of participants completed all training sessions, further indicating implementation of the clinical study is feasible. COVID-19 presented challenges both with exercise training and outcome measurement collection. Remote exercise training was minimally adjusted from the original standardised protocol to enable the research to continue (at home instead of a local gym). During COVID-19 outbreaks local gyms were closed and research recruits were not allowed to attend the hospital outpatient gyms due to restrictions. As a result, remote training was continued at home without gym attendance utilising variations to exercises individualised to equipment available and resistance exercise prescription continued at a load rate of RPE of 6–7 with each exercise. If participants were unable to achieve the exercise load completed at the gym they were instructed to increase the repetitions and work to a RPE of 6–7. As the study continued, those who were recruited later were also offered remote training at home, not a gym, with the aim to increase recruitment rates and adherence to the long duration protocol. The use of PhysiApp® increased the ease of transition to remote training as it included the exercise program with photos and videos. The smartphone app was easily accessible and enabled remote monitoring by the researcher through patient diarising. The app may also have contributed to improved adherence to training as it facilitated sessions what would otherwise had to be cancelled or rescheduled. It has been demonstrated that physical activity apps can improve health outcomes, and adherence to the app is improved if combined with external monitoring, as was implemented in this study [26, 27]. That said, there were technical difficulties with Physitrack® for ten days during the study where individuals were unable to diarise any exercises completed. This affected two participants. Both individuals were proactive and kept a written record of their exercise training during this time. The flexibility with exercise training allowed the study to continue without further delays during the COVID-19 pandemic. Other sites throughout the world have implemented virtual research assessments or follow ups when able to, to tackle the challenges of completing research during COVID-19 restrictions [28]. However, we acknowledge, for the ease of replication for further studies, implementation of a standardised exercise protocol would be best for remote home and/or gym training.

At the inception of this study, we proposed to collect data from 20 eligible participants within 12 months. The project was initially delayed by six months due to the COVID-19 pandemic as the early restrictions implemented in Western Australia prevented research outpatients attending the hospital. Thus, conducting outcome assessments and exercise prescription were not feasible and could not be completed. Once restrictions were eased 21 participants consented over six months. Recruitment had its challenges as only eight percent (8%) of the screened participants meeting the inclusion criteria were either eligible to participate, primarily due to living outside the Greater Perth region, or were contactable. The final recruitment rate was 49% and included those eligible and who agreed to participate. Frequent reasons for refusal were personal commitments or being time poor which likely reflects the median age of participants, 41 (IQR 28–58) years, who were of working and family commitment age. Six participants withdrew (four from Con-Ex and two from Ex-Con, Fig 1) due to injury, personal issues and direct impact of introduced COVID-19 restrictions during their study period. One of these, participant six, was initially recruited and randomised into the control arm of the study. He completed two testing sessions across the first three weeks of the study period before he had to withdraw due to an upcoming surgery that rendered him ineligible. Three months after his surgery he met the eligibility criteria and was re-enrolled to the study as participant 21. Due to his recent surgery, he was required to partake in supervised exercise training for the experimental period of the study. Participants in the Exercise—Control group were offered a free six week block of exercise training after completion of the study as an incentive to continue in the study. Two participants accepted this offer. Parking tickets were also provided to all participants to limit the cost and burden to each participant. The cross over study design in this study was feasible, however it could be argued that having the exercise intervention first could improve retention rates as only two withdrew comparative to four when the exercise intervention was second. Despite the difficulty of recruitment, the eligibility criteria were feasible and suitable.

Anecdotally, participants were enthusiastic with the study and had no complaints with the outcome measurement battery. Several participants provided specific feedback. “The exercise program was good. It was motivating and all the staff supervising was professional. It was completed remotely just as well as the physio app allowed tracking of the completion of the exercises by the supervisor and the remote exercises could be completed with no special equipment. I was just happy to participate in a very small way to the research”. One participant preferred the gym over remote training due to the support and encouragement by the researchers, however found the use of PhysiApp® kept them on track with the training program during COVID-19 lockdown.

### Limitations

The limited timeline for completion of the study resulted in the number of participants completing the study not meeting the pre-planned target of 20. However, in its current form the numbers are large enough to determine the feasibility of the study. Ideally, moving forward larger studies would be beneficial to improve the power of analysis of assessing the effect of exercise on burn induced inflammation.

Researcher time was funded, and all aspects of the study were carried out in non-clinical time. Limited funding contributed to the available timeline but also enabled the study to be completed.

## Conclusion

This study demonstrates the feasibility of conducting a long duration exercise training study in individuals greater than a year after burn injury The knowledge gained helps improve the methodology in larger-scale projects. It demonstrates how clinical projects despite the COVID-19 pandemic could continue with minimal protocol flexibility and adaptation of individuals and practices whilst keeping participants and staff safe.

## Supporting information

S1 Checklist CONSORT 2010 checklist of information to include when reporting a randomised trial*. (DOC)

S1 Dataset Minimal dataset. (CSV)

S1 File Study protocol. (DOCX)

## Abbreviations

IPAQ: International Physical Activity Questionnaire
IQR: inter quartile range
LLFI: lower limb functional Index
PHQ: patient health questionnaire
QDASH: Quick Disability of arm, shoulder and hand
TBSA: total body surface area
CON-EX: control-exercise condition
EX-CON: exercise-control condition
RPE: rating of perceived exertion
T1-5: time point 1–5
