# Supplementary material for: Does exercise influence burn-induced inflammation: A cross-over randomised controlled feasibility trial
Source: PLoS One. 2022 Apr 1;17(4):e0266400. doi: 10.1371/journal.pone.0266400 (PMC8974991; doi:10.1371/journal.pone.0266400)
Supplement: S1 File — (DOCX) [file pone.0266400.s003.docx]

Does exercise influence burn induced inflammation?

Contents

[1. Administrative Information 3](#_Toc26429918)

[1.1. Trial Name: 3](#_Toc26429919)

[1.2. Trial Short Name 3](#_Toc26429920)

[1.3. Trial Registration 3](#_Toc26429921)

[1.4. Protocol Version / Date 3](#_Toc26429922)

[1.5. Project Record Number 3](#_Toc26429923)

[1.6. Funding Sources 4](#_Toc26429924)

[1.7. Protocol Contributors 4](#_Toc26429925)

[1.8. Role of funding bodies 5](#_Toc26429926)

[2. Introduction 6](#_Toc26429927)

[2.1. Background and Rationale 6](#_Toc26429928)

[2.2. Primary Objective 9](#_Toc26429929)

[2.3. Trial Design 9](#_Toc26429930)

[3. Methods: Participants, interventions and outcomes 9](#_Toc26429931)

[3.1. Study Setting 9](#_Toc26429932)

[3.2. Eligibility Criteria 9](#_Toc26429933)

[3.3. Study Interventions 10](#_Toc26429934)

[3.4. Criteria for discontinuing or modifying allocated interventions 11](#_Toc26429935)

[3.5. Strategies to improve adherence to protocols 11](#_Toc26429936)

[3.6. Outcome Measures 11](#_Toc26429937)

[3.7. Participant Timeline 12](#_Toc26429938)

[3.8. Sample Size 13](#_Toc26429939)

[3.9. Recruitment 13](#_Toc26429940)

[4. Methods: Data collection, management and analysis 13](#_Toc26429941)

[4.1. Data collection Methods 13](#_Toc26429942)

[4.2. Data Management 14](#_Toc26429943)

[4.3. Statistical Analysis 14](#_Toc26429944)

[5. Methods: Monitoring 14](#_Toc26429945)

[5.1. Data monitoring and harms 14](#_Toc26429946)

[6. Ethics and dissemination 15](#_Toc26429947)

[6.1. Research ethics approval 15](#_Toc26429948)

[6.2. Protocol amendments 15](#_Toc26429949)

[6.3. Consent 15](#_Toc26429950)

[6.4. Confidentiality 16](#_Toc26429951)

[6.5. Declaration of interests 16](#_Toc26429952)

[6.6. Access of data 16](#_Toc26429953)

[6.7. Dissemination policy 16](#_Toc26429954)

[7. References 17](#_Toc26429955)

# Administrative Information

## Trial Name:

Does exercise influence burn induced inflammation?

## Trial Registration

ACTRN12620000237987p

## Protocol Version / Date

V1 160819

## Project Record Number

RGS0000003381

## Funding Sources

Fiona Wood Foundation $70,000.00

## Protocol Contributors

**Coordinating Principal Investigator**: Dr Pippa Kenworthy

State Adult Burns Unit, Fiona Stanley Hospital

Senior Physiotherapist, PhD, Fiona Stanley Hospital

Role in this project: Project co-ordinator, study design and reporting, including funding acquittal.

Telephone: 616 72926

Email: [Pippa.Kenworthy@health.wa.gov.au](mailto:Pippa.Kenworthy@health.wa.gov.au)

**Principal Investigator 1**: Prof Fiona Wood

Head of Department, State Adult Burns Unit, Fiona Stanley Hospital

University of Western Australia

Role in this project: Assist with protocol development, recruitment, data interpretation and preparation and editing of reports and manuscripts.

**Principal Investigator 2**: Dr Dale Edgar

State Adult Burns Unit, Fiona Stanley Hospital / University of Notre Dame

Role in this project: Assist with coordination of study; independent assessor where necessary; preparation and editing of reports and manuscripts.

**Principal Investigator 3**: Dr Mark Fear

Burn Injury Research Unit, University of Western Australia

Role in this project: Senior Research Fellow, Microbiome laboratory analysis, editing of reports and manuscripts.

## Role of funding bodies

The funder, FWF, remains at arm’s length and have no specific input or authority over the following activities which remain the responsibility of the study investigators: study design; collection, management analysis and interpretation of data; writing of the report or decision to submit for publication.

# Introduction

Participants with a ≥ 5%TBSA burn injury, ≥ 1year after burn injury will be recruited. All patient demographic (gender, age, PMHx); injury or intervention (surgical procedures); and system (LOS, complications) co-variates will be recorded at recruitment.
The primary aim of the study will be to longitudinally analyse the effect a 6-week pulse of exercise has on the level of inflammatory biomarkers including TNF alpha and a multiplex of other cytokines at different times frames after burn. A randomised cross-over trial will be conducted with participants randomised to either an exercise or control condition in the first phase. In the second phase participants will cross over into the alternate condition e.g phase 1 exercise condition participants will change over to the control condition in phase 2. Outcome measurement and blood sampling time points will be pre-intervention, at 3 weeks after commencement of intervention/control and then at 6 weeks (completion of study period 1). These will then be repeated in study period 2 at the same time points.

## Background and Rationale

Unlike other pathologies, injury can happen *to anyone, at any time*. Treatments to improve recovery after injury are imperative now. Evidence suggests that exercise directly influences inflammation and immune function. Unlike other treatments, exercise is accessible to all regardless of socioeconomic status or available facilities. The enormity of the human and fiscal costs of injury and surgery, consequent morbidity and loss of productivity is difficult to fathom or quantify accurately (Unpub PI1- FW). By quantifying the relative levels of chronic systemic inflammation from a burn injury this study may also provide new evidence to expose the mechanisms linking biomarkers to long term disability.

We plan to facilitate an analysis of the chronic systemic pathophysiology of the patient ≥1 year after burn injury with specific reference to inflammation and to understand the influence of exercise, if any, after burn injury measured using biochemical, haematological and cellular responses over time. In the timeframe available, the primary aim of this study is to pilot the cross over trial and determine if exercise alters inflammation after burn injury as measured by inflammatory biomarkers (eg TNFα, cortisol, catecholamines and cytokines) across time.

Mapping of the inflammatory ‘recovery’ trajectories after burn injury, and applying an exercise intervention, will provide novel insight into the optimal timing of treatments to improve patient outcomes. With this new knowledge, clinicians can aspire to rethink the implications of chronic inflammation and adjust the education and interventions to reduce latent consequences and health costs post-injury. Finally, understanding if exercise alters longitudinal chronic inflammatory pathways will inform and improve future studies to determine if exercise may reduce the long-term risk of developing systemic diseases of ageing and cancer.

Latent Impact of Injury on Health

Burn trauma is one of the top five causes of injury across the globe. It is undeniable that burn and trauma survivors endure temporary and permanent disabilities (1). The health, work productivity and patient costs due to transient and long term impairments are enormous and complex to quantify (WHO Report, pg7) (2, 3). Further, uniquely identifiable, a burn injury changes the life trajectory of survivors by a mechanism, as yet, to be clearly understood (4, 5). After burn, a prolonged and extreme inflammatory, catabolic response occurs (6, 7). The inflammatory load after burn injury is implicated as the primary driver of increased risk of mortality (8); systemic diseases (9, 10) and cancer (11). However, not only burn but also non-burn injury is now linked to development of, and hospitalisation for cardiovascular disease and diabetes (12, 13). The common feature that challenges the acute and long-term immune function and physical recovery after bodily injury is the inflammatory load. The interactions of inflammatory and immune biomarkers with meaningful health recovery measures have never been quantified. Due to the development of the Australian National Phenomics Centre (ANPC) on the site of Harry Perkins Institute (co-located with Fiona Stanley Hospital), we are in a position to handle and hold on to samples than ever possible.

Immune Response to Injury

The inflammatory or immune response, marked by systemic cytokine and chemokine release, is initiated by burn injury (14, 15) and tissue damage (7) and leads to a depression of the immune system in response. If episodes of inflammation (eg infection) are repeated, it stands to reason that the capacity of the host immune system to respond to said stresses, is reduced over time (16, 17). In the burns population, current coinvestigators demonstrated emphatically that the inflammatory process has a chronic systemic effect, which is associated with lifelong consequences (10-12, 18), postulated to be mediated by the impact of inflammation on the immune system (19). In addition, the impaired function of the immune system is implicated when considering the tolerance of human hypertrophic burn scar. Scar is abnormal in its architecture, genetic expression, cell phenotype and chemistry (20). The host tolerance, and growth in children, of the scar construct is an indication that the immune surveillance is altered and permissive for life. Could there be a ‘hole’ created in the immune ‘net’ due to an inflammatory insult from burns? After burn injury an elevated pro-inflammatory environment has been demonstrated in paediatrics up to three years after injury (Unpub data AI –MF, AI FW) (21).

Impact of Exercise on inflammation

In cancer surgery populations, the lack of pre-morbid muscle mass, or sarcopenia, is associated with increased mortality and post-op complications and further, predicted long term survival rates (22). Exercise, in varying modes and patient populations, is being increasingly used in clinical rehabilitation settings to improve or restore muscle mass; physical and psychological outcomes; and, quality of life and wellbeing (23-25). Exercise during acute (Unpub data, PI2 DE, PI1 FW) and sub-acute post-burn periods is feasible, beneficial and safe (26, 27). Thus, exercise is an accessible, low cost treatment that may ameliorate immune depression and the negative long term consequences. However, it is unknown who will respond to exercise or exactly by what mechanism exercise influences inflammation.

Preliminary Data (informing methods)

Exercise Reducing Inflammation

Previous research led demonstrated his capacity to complete supervised training programs and suggested the positive influence of exercise on bodily inflammation (28-31). These investigations in prostate cancer patients demonstrated the role of exercise in specifically reducing cardiovascular disease risk factors and confirming exercise and measurement methodologies to be applied in this study. The similar intervention as proposed in this study (aerobic and resistance exercise) resulted in significant favourable adaptations in cardiorespiratory capacity (5%), fat oxidation (17%) and body composition (3.8%) when compared to usual care (28).

Inflammatory Biomarker

The extensive research of the Galveston group catalogues the hypermetabolic, inflammatory and immune responses and targeted interventions after burn injury (16, 32, 33). Clinical research, led by PI1 FW, using ablative fractional CO2 laser (AFCO2L) as a promising scar treatment has led to a greater understanding of the neuroinflammatory biomarkers specific to skin injury and hypertrophic scarring eg Calcitonin Gene Related Peptide (CGRP) (34). Further research headed by PI3 MF and PI1 FW demonstrated cytokines TNFa, IL-2, IL-7 and IFNg were all significantly elevated at least two years after burn injury in children. Jescke et al (2011) also observed elevated levels for almost all cytokines in children three years after burn injury when compared to non-burn patients (35). Further, exercise studies in acute adult burn patients (TBSA >5%) at Fiona Stanley Hospital investigated the relationship of C-reactive protein (CRP) overtime in both the exercise and control group. They also paired serum C-reactive protein (CRP) with SF36 and Burn Specific Health Scale-Brief (BSHS-B) surveys at 6, 12 and 26 weeks post-burn (n=19pts, P.Gittings unpub data, 2018). This pilot data indicated that the inflammatory marker (CRP) is significantly and negatively associated with quality of life physical domains (β=-0.02 to -0.51, P<0.001), in minor to moderate TBSA burns. Additionally, participants in the intervention group tended towards a faster reduction in CRP compared to control patients (p<0.002). It has therefore been demonstrated that chronic long term inflammation is present after non-severe burn injury.

Outcome measures

We plan to facilitate an analysis of the systemic pathophysiology of the patient after burn injury with specific reference to inflammation and to understand the role of exercise after burn injury measured using biochemical, haematological and cellular responses over time.

## Primary Objective

The primary objective of this trial is to determine if exercise alters inflammation after burn injury as measured by inflammatory biomarkers (primarily TNFα) across time.

## Trial Design

This clinical trial is a single-centre, cross-sectional, crossover study designed to examine if the inflammation is altered by a pulse of exercise applied as the intervention. We hypothesise a pulse of exercise will reduce long term inflammatory load after burn injury. Participants, from each year group (time after injury - 1-3 yrs, 3-5yrs, > 5yrs (± 3 months)), will be block randomised by year after burn to either the exercise or control condition in the first study period. The exercise intervention group will complete a six-week gym program including both resistance and aerobic exercise, three days per week.

# Methods: Participants, interventions and outcomes

## **Study Setting**

The study will be conducted in the State Adult Burns Unit outpatient gym at Fiona Stanley Hospital with some exercise sessions being undertaken at participant’s local gym.

## Eligibility Criteria

Participants that meet the inclusion criteria will be identified and recruited from the SABU database. All patient demographic (gender, age, PMHx); injury or intervention (surgical procedures); and system (LOS, complications) co-variates will be recorded at recruitment.

*Inclusion Criteria*

- ≥ 5%TBSA burn injury
- Time post injury ≥ 1 year
- Male or female, ≥ 18 years old
- Satisfactory self-described health status (excluding burn injury)
- Subject understands the proposed project, is able to consent to participation

Exclusion Criteria

- Acquired or pre-existing neurological injury or disease conditions which influence the capacity to complete future exercise or walking program eg nerve injury or multi-trauma; spinal cord injury, CVA / central nervous system lesions; Patients with no fixed address.
- Unstable cardiac conditions
- Intellectually challenged patients
- Non – English speaking
- Pregnant Women
- Cancer

## Study Interventions

Control condition: The control group will be asked to continue on with normal daily tasks and their normal activity levels, monitored by activity trackers. Participants will participate in the same 6-week intervention condition (cross over study).

Exercise Intervention condition: These participants will train three times per week for six weeks either in a supervised exercise session (18 sessions) at FSH or at their local gym. The sessions will last ~60 minutes including warm up, resistance exercises, cardiovascular exercise and cool down periods. Sessions will begin with a 5-minute dynamic warm up at a light-moderate intensity equating to an RPE of 11-12 on the Borg Scale. The aerobic warm up modality will vary from session to session and include the use of treadmill and cycle ergometers. Participants will be instructed to complete an equal amount of sessions on each of these. Participants will then move into 20 minutes of aerobic exercise (AE) utilizing a variety of exercise modes and equipment. Intensity for the AE will be prescribed at 70-90% of age predicted HR max with the average HR recorded at the end of each aerobic session. The resistance training (RT) regime will consist of six exercises targeting upper and lower major muscle groups. This intervention has been used previously in a large scale clinical population study (36, 37). The RT intensity will be set at 3 sets of 10 repetitions. The resistance exercises will be progressive in nature whereby if a participant can perform an additional 2 repetitions in the final set for 2 consecutive sessions, the resistance will be increased by 5%. This progressive overload approach is widely used in resistance training programming to maximize the level of adaptation (38). The session will conclude with a 5-minute cool down period incorporating low level aerobic activities and stretching. In addition to the gym sessions, participants will be requested to perform an additional 90 minutes of home-based moderate intensity aerobic exercise (in the form of walking). The combination of 60 minutes gym based aerobic exercise and 90 minutes home based aerobic exercise is designed to meet with ACSM/AHA association guidelines of 150 minutes aerobic exercise per week (39). This exercise regime has been designed to elicit the optimal cardiorespiratory and neuromuscular response in the 6-week time-frame allocated whilst maximizing adherence and retention. An accredited Physiotherapist will be responsible for each supervised exercise session. For participants to undertake exercise sessions at their local gym they will need to be assessed as ‘low risk of adverse events to exercise’. This will be determined by an adult pre-exercise risk stratification assessment which is the Patient Health (APOC) questionnaire.

Feasibility*:* Participants undertaking exercises at their local gym will be, required to attend FSH at least once per week to enable appropriate progression of their exercises. Where this is not possible they will need to make contact with the supervising physiotherapist via phone to support appropriate progression of their exercise program at least weekly. The free Physitrack app will also be utilised as an exercise diary for participants to record, in real time, the individual exercise sessions completed. The exercises and exercise prescription is also detailed in the app for participants to refer to. The supervising therapist (PI) has access to individual’s account providing another platform to engage with the participants and manage their exercise progression. At a minimum, participants will need to be able to attend FSH for baseline measures, at the mid-way point (three weeks), and at completion of program (6 weeks) for data collection.  Activity trackers will be utilised for both groups to quantify their average weekly activity. Participants will be provided with an activity tracker (either wrist worn or pedometer style (clothes worn)) if they do not own one themselves. They will need to be worn, at a minimum, every waking hour.

## Criteria for discontinuing or modifying allocated interventions

Exercise prescription may be modified (from the described prescription and progression) based on the participant’s response to the exercise. Intervention will be discontinued if the patient is no longer able to participate in exercise safely due to injury or a medical issue.

## Strategies to improve adherence to protocols

An accredited Physiotherapist will be responsible for each supervised exercise session at FSH to facilitate adherence and ensure safe exercise practices and techniques are adhered to.

Participants undertaking exercises at their local gym will be required to attend FSH once per week to enable appropriate progression of their exercises. Where this is not possible they will need to make contact with the supervising physiotherapist via phone to support appropriate progression of their exercise program. At a minimum, participants will need to be able to attend FSH for baseline measures, at the mid-way point (3 weeks) and at completion of program for data collection (for both study period 1 and 2). General activity will be monitored throughout the entirety of the study (including the washout period) using wearable devices. This will enable us to track participants ‘general’ activity.

## Safety Processes

Should any adverse event occur in the hospital (FSH) then the patient will be provided with first aid and then assisted to ED for assessment and treatment as necessary. Participants deemed ‘low risk’ with the APOC questionnaire and wishing to perform their exercise program at a local gym will be asked to confirm the public insurances in place, and the safety procedures followed at the gym if they suffer an adverse event. The investigator will confirm with the patient that they have reviewed these assurances prior to prescribing their program closer to home. If any adverse events occur while in the local gym then the procedure will be that the patient first seeks first aid from gym staff and then attends their local GP or ED as soon as possible. Treatment and, or adjustment of the exercise program will be made accordingly if required, as per medical review. Participants will also have access to investigators by phone to seek guidance if unsure. Potential for injury with exercise however will be minimised by having the initial exercise prescription and exercise session completed by an accredited physiotherapist. Progressions of exercises are also made under the guidance of an accredited physiotherapist by phone or the physitrack app. Further, we will ensure the gym facility to be attended has supplied adequate evidence of public liability.

## Outcome Measures

To provide greater understanding of the impact of exercise after burn injury a battery of routinely collected physical, functional and psychological outcomes, will be measured.

Primary Outcome

*TNF alpha* will be collected at baseline, mid-way and post intervention for study periods 1 and 2 (Figure 1).

Secondary Outcomes (Table 1)

The clinical outcomes to be collected include physiological (non-invasive muscle mass bioimpedance spectroscopy (40, 41)); physical (isometric grip and muscle strength (42); fitness - modified Chester Step Test [unpub data, F. Coll, D.W. Edgar, K. Hill]); functional (QuickDASH (43), LLFI (44)); neurological (Semmes-weistein monofilament, two-point discrimination, proprioception) (45); heat response and thermoregulation (optical sensitive coherence topography, core temperature, heart rate) (46, 47); metabolome analysis (urine, skin and hair samples, plasma (multiplex of other cytokines)); microbiome analysis (skin and rectal swabs) and, quality of life (SF36 (48)). In addition, we will include a self-reported pre-study activity survey (International Physical Activity Questionnaire (IPAQ)) to ensure that the exercise levels of all recruited patients are accounted for.

**Table 1:** **Outcome measures** - All measures are collected at baseline (0 weeks), mid-way (3 weeks), post intervention (6 weeks) in study periods 1 and 2.

| **Outcome Measures** | |
| --- | --- |
| Inflammatory Biomarker | *TNF alpha* |
| Physiological and Physical | Bioimpedance Spectroscopy  Grip strength  Muscle Strength |
| Fitness/activity | Modified Chester step test,  International Physical Activity Questionnaire  Wearable fitness tracker data |
| Functional (subjective) | Quick Dash  Lower limb functional Index |
| Neurological | Semmes-weistein monofilament  Two-point discrimination  Proprioception |
| Metabolome analysis | Plasma (multiplex of other cytokines), urine, skin and hair |
| Microbiome analysis | Rectal skin swabs |
| Quality of Life | SF36 |

## Participant Timeline

Each participant (n=20) will be asked to commit to a total of 16 weeks: 6 weeks for both the intervention and control group, with a 4 week break between the two (Figure 1). We anticipate the trial (n=20) to be completed within a year. This includes recruitment, finalisation of the intervention and analysis.

Outcome measurement and blood sampling time points will be pre-intervention, at 3 weeks after commencement of intervention/control and then at 6 weeks (completion of study period one). These will then be repeated in study period two (Figure 1).

|  | **Study Period 1**  **6 weeks** | **Cross-over**  **4 week rest period** | **Study Period 2**  **6 weeks** |  |
| --- | --- | --- | --- | --- |
|  | Exercise Condition |  | Exercise Condition |  |
| Recruitment/  informed  consent |  |  |  |  |
| (over 12 months) | Control Condition |  | Control Condition |  |
|  |  |  |  |  |
|  | **Baseline** | **Outcome Measures Timeline**  **Mid-way** | **Post intervention** |  |
|  | 0 weeks | 3 weeks | 6 weeks |  |

Figure 1: Participant timeline

## Sample Size

This is a pilot and feasibility study planned to be completed in the FWF fellowship period. Current assumptions to estimate the sample size for this study, relates to data available from burn patient chronic TNF alpha levels, which were used to inform the assumptions of the calculation. A sample size of 85 patients (allowing for 15% loss to follow up) would be required to achieve 80% power to detect a change in TNF alpha, with alpha set at 5% and with an effect size of 0.3. With this pilot data, we will aim to hone the assumptions relating to the sample size to achieve definitive study outcomes and improve the strength of future grant applications to fund other components of this trial.

## Recruitment

Patients found in the SABU database, that fit the inclusion criteria will be telephoned and invited to participate in the trial. The research project will be advertised on the Fiona Wood Foundation website and face book page to capture any past burns patients not on the SABU database and children that have been burnt who are now adults. A preliminary review of SABU census to date predicted the number of patients eligible for recruitment at least one year after burn is 139. It is feasible that a significant proportion of the total of 139 patients could be contacted to offer recruitment.

# Methods: Data collection, management and analysis

## Data collection Methods

All patient demographic (gender, age, PMHx); injury or intervention (surgical procedures); and system (LOS, complications) co-variates will be recorded at recruitment and collected from the burns information management system (BIMS).

TNF alpha

A 4mL sample of blood will be collected into one Lithium Heparin (no gel, purple vile) tubes and then separated into aliquots for a) *TFN alpha* assay; and, b) other cytokine assays (UWA Burn Injury Research Unit (BIRU)). All samples will be prepared in a standardised method, separated into the required aliquots and stored in a -80^o^ freezer within 2 hours of collection. The preparation of the blood samples are as per PBMC Isolation Protocol.

The FSH Burn Unit has a suitable freezer for short term storage (< 1 week) at which time, the samples will be moved for long term storage and, or processing at UWA BIRU (inflammatory cytokine assay) to facilitate batch analyses and minimise costs. Storage of the samples at UWA BIRU are ‘in-kind’. Any blood samples not utilised will be stored, in-kind at UWA BIRU, for future transcriptomic and metabolomic analysis (within 10 years of this study).

## Data Management

The digital/soft copy raw data (outcome measures) and participant details for each patient are stored, unidentified, in a locked folder on the FSH State Adult Burns Unit portion of the secure WA health W: drive. The subfolder and file will be password protected. The passwords will be changed regularly and an automatic screen lock will be placed at 5 minutes. Fiona Stanley Hospital has a complex arrangement of firewalls and other security measures that prevent access from external sites. The participants (identified by numbers) original hard copy data sheets (including details and measures) are stored in a locked filing cabinet behind swipe access in Level 4B Burns Outpatients. The data will be stored for ten years, as required by law.

## Statistical Analysis

The primary aim of this clinical trial is to explore if a pulse of exercise influences the inflammatory biomarkers (TNFa, IL-2, IL-7 and IFNg), after burn injury.

Biomarker (serum sample) levels will be analysed using linear mixed models, including fixed effects for time, condition and their interaction, with a random effect for participant to account for the correlated observations.  Preliminary analyses will consider whether order of treatment influenced the outcome measures, and this factor will be included in the final model if appropriate. The influence of potential confounding factors (e.g. age, time post injury) will also be assessed.

Confounders such as self-motivated exercise activity will be recorded at each assessment time point to allow adjustment in the final analyses.

# Methods: Monitoring

## Data monitoring and harms

Adverse events will be recorded within the electronic storage database.

Risks

- Blood sampling: invasive
- Potential for injury with exercise however these will be minimised with supervised sessions from an accredited physiotherapist or regular contact with an accredited physiotherapist if exercise sessions completed locally to the patient.
- Post exercise delayed onset muscle soreness – this will be minimised through appropriate exercise prescription from an accredited physiotherapist and made clear to the participants it may be a side effect and will be minimised by conservative starting points with exercise and slow progression at 5% instead of 10% which would be proposed for the uninjured population.

Cardiac events, persisting dizziness (cardio vascular / glycaemic control events), injury due to weights or equipment. For these reasons a will supervise all sessions completed in the hospital. Should any adverse event occur in the hospital then the patient will be provided with first aid and then assisted to ED for assessment and treatment as necessary. The patient outside the hospital will also have access to investigators by phone. If any adverse events occur while in the local gym then the procedure will be that the patient attends their local GP or ED as soon as possible.

Benefits

- Benefits of exercises: maintenance or improved muscle mass, improved cardiovascular fitness
- Externally motivated to perform exercise

# Ethics and dissemination

## Research ethics approval

The study and protocol will be submitted for full review by the South Metropolitan Health Service Human Research Ethics Committee (SMHS HREC).

## Protocol amendments

Protocol amendments will be submitted for review by the SMHS HREC. Once approved they will be communicated in person by the CPI to the other investigators.

## Consent

All patients meeting the eligibility criteria will be provided a participant information and consent form. They will be given the opportunity to ask questions about the study and will be encouraged to discuss enrolment with family or friends. It will be emphasised that they are free to withdraw from the trial at any time without consequence. As highlighted in the participant information sheets, all study participants will be given the opportunity to decline any particular component or sampling method. Participants will be considered enrolled in the study after signing the consent form, witnessed by a study clinician.

The standard consent will include consent to storage of data and biological samples for extended study i.e. further / continued research related to the study focus of inflammation in chronic burn injury. Participants will not be asked to consent to unspecified research or to genetic research.

## Confidentiality

Participants will be assigned a unique study number on enrolment that will be used as the only participant identifier in the study dataset. No identifying data (unique medical record number, name, date of birth, address) will be captured in the study dataset.

Biological samples will initially be labelled with both the standard hospital addressograph and a label to identify the participant's study number. After primary processing (within hours of collection), sample aliquots will no longer have an addressograph and will be labelled with unique sample number and the participant's study number only. As such, processed samples will be re-identifiable only to investigators with access to the participant log. Only processed samples with identifiers removed will be put into storage or transferred for analysis at another facility.

## Declaration of interests

All study investigators have confirmed that they do not have any financial or other conflicts of interest to declare in relation to this study.

## Access of data

Only investigators that are Department of Health employees will access or have access to the individually identifiable data used and collected in this project. Such access will be in line with their routine clinical duties. Investigators outside of the Department of Health will have access to the final study dataset with identification only by unique study number.

## Dissemination policy

Trial results will be made available to participants through publication on the Fiona Wood Foundation website. It is anticipated that trial results will be made publicly available through a series of peer reviewed journal articles.

# References

1. Wasiak J, Lee SJ, Paul E, Mahar P, Pfitzer B, Spinks A, et al. Predictors of health status and health-related quality of life 12 months after severe burn. Burns. 2014.

2. World Health Organisation U. Injuries and Violence: The Facts. Geneva, Switzerland; 2014. Contract No.: ISBN 978 92 4 156357 4.

3. Organisation WH. WHO Guide to identifying the economic consequences of disease and injury. Geneva, Switzerland: WHO Press, World Health Organization; 2009.

4. Swanson JW, Otto AM, Gibran NS, Klein MB, Kramer CB, Heimbach DM, et al. Trajectories to death in patients with burn injury. The journal of trauma and acute care surgery. 2013;74(1):282-8.

5. Duke JM, Rea S, Boyd JH, Randall SM, Wood FM. Mortality after burn injury in children: a 33-year population-based study. Pediatrics. 2015;135(4):e903-10.

6. Finnerty CC, Herndon DN, Przkora R, Pereira CT, Oliveira HM, Queiroz DM, et al. Cytokine expression profile over time in severely burned pediatric patients. Shock (Augusta, Ga). 2006;26(1):13-9.

7. Vanzant EL, Lopez CM, Ozrazgat-Baslanti T, Ungaro R, Davis R, Cuenca AG, et al. Persistent inflammation, immunosuppression, and catabolism syndrome after severe blunt trauma. The journal of trauma and acute care surgery. 2014;76(1):21-9; discussion 9-30.

8. Duke JM, Boyd JH, Randall SM, Wood FM. Long term mortality in a population-based cohort of adolescents, and young and middle-aged adults with burn injury in Western Australia: A 33-year study. Accid Anal Prev. 2015;85:118-24.

9. Randall SM, Fear MW, Wood FM, Rea S, Boyd JH, Duke JM. Long-term musculoskeletal morbidity after adult burn injury: a population-based cohort study. BMJ Open. 2015;5(9):e009395.

10. Fear VS, Boyd JH, Rea S, Wood FM, Duke JM, Fear MW. Burn Injury Leads to Increased Long-Term Susceptibility to Respiratory Infection in both Mouse Models and Population Studies. PloS one. 2017;12(1):e0169302.

11. Duke JM, Bauer J, Fear MW, Rea S, Wood FM, Boyd J. Burn injury, gender and cancer risk: population-based cohort study using data from Scotland and Western Australia. BMJ Open. 2014;4(1):e003845.

12. Duke JM, Randall SM, Fear MW, O'Halloran E, Boyd JH, Rea S, et al. Long term cardiovascular impacts after burn and non-burn trauma: A comparative population-based study. Burns. 2017;43(8):1662-72.

13. Duke JM, Randall SM, Fear MW, Boyd JH, Rea S, Wood FM. Diabetes mellitus after injury in burn and non-burned patients: A population based retrospective cohort study. Burns. 2018.

14. Finnerty CC, Jeschke MG, Herndon DN, Gamelli R, Gibran N, Klein M, et al. Temporal cytokine profiles in severely burned patients: a comparison of adults and children. Mol Med. 2008;14(9-10):553-60.

15. Kowal-Vern A, Sharp-Pucci MM, Walenga JM, Dries DJ, Gamelli RL. Trauma and thermal injury: comparison of hemostatic and cytokine changes in the acute phase of injury. The Journal of trauma. 1998;44(2):325-9.

16. Finnerty CC, Herndon DN, Chinkes DL, Jeschke MG. Serum cytokine differences in severely burned children with and without sepsis. Shock (Augusta, Ga). 2007;27(1):4-9.

17. Cohen MJ, Carroll C, He LK, Muthu K, Gamelli RL, Jones SB, et al. Severity of burn injury and sepsis determines the cytokine responses of bone marrow progenitor-derived macrophages. The Journal of trauma. 2007;62(4):858-67.

18. Duke J, Rea S, Semmens J, Edgar DW, Wood F. Burn and cancer risk: a state-wide longitudinal analysis. Burns. 2012;38(3):340-7.

19. Tuan TC, Hsu TG, Fong MC, Hsu CF, Tsai KK, Lee CY, et al. Deleterious effects of short-term, high-intensity exercise on immune function: evidence from leucocyte mitochondrial alterations and apoptosis. Br J Sports Med. 2008;42(1):11-5.

20. Stevenson A. Investigating the role of epigenetics in scar maintenance. Perth: University of Western Australia; 2016.

21. Jeschke M, Gauglitz G, Kulp G, Finnerty C, Williams F, Kraft R, et al. Long-Term Persistance of the Pathophysiologic Response to Severe Burn Injury. PloS one. 2011;6(7):e21245.

22. Jones K, Gordon-Weeks A, Coleman C, Silva M. Radiologically Determined Sarcopenia Predicts Morbidity and Mortality Following Abdominal Surgery: A Systematic Review and Meta-Analysis. World J Surg. 2017;41(9):2266-79.

23. Cucuzzo NA, Ferrando A, Herndon DN. The effects of exercise programming vs traditional outpatient therapy in the rehabilitation of severely burned children. The Journal of burn care & rehabilitation. 2001;22(3):214-20.

24. Kadaglou NPE, Iliadis F, Angelopoulou N, al e. The anti-inflammatory effects of exercise training in patients with type 2 diabetes mellitus. *Eur J Cardiovasc Prev Rehabil* 2007;14:837-43

25. Schneider JK, Cook JH, Jr., Luke DA. Cognitive-behavioral therapy, exercise, and older adults' quality of life. West J Nurs Res. 2008;30(6):704-23.

26. Suman OE, Herndon DN. Effects of cessation of a structured and supervised exercise conditioning program on lean mass and muscle strength in severely burned children. Archives of Physical Medicine & Rehabilitation. 2007;88(12 Suppl 2):S24-9.

27. Paratz JD, Stockton K, Plaza A, Muller M, Boots RJ. Intensive exercise after thermal injury improves physical, functional, and psychological outcomes. The journal of trauma and acute care surgery. 2012;73(1):186-94.

28. Wall AB, Galvão AD, Fatehee RN, Taaffe JD, Spry UN, Joseph UD, et al. Exercise Improves V˙O2max and Body Composition in Androgen Deprivation Therapy–treated Prostate Cancer Patients. Medicine & Science in Sports & Exercise. 2017;49(8):1503-10.

29. Taaffe DR, Newton RU, Spry N, Joseph D, Chambers SK, Gardiner RA, et al. Effects of Different Exercise Modalities on Fatigue in Prostate Cancer Patients Undergoing Androgen Deprivation Therapy: A Year-long Randomised Controlled Trial. European Urology. 2017;72(2):293-9.

30. Wall AB, Galvão AD, Fatehee RN, Taaffe UD, Spry UN, Joseph UD, et al. Maximal Exercise Testing of Men with Prostate Cancer Being Treated with Androgen Deprivation Therapy. Medicine & Science in Sports & Exercise. 2014;46(12):2210-5.

31. Wall B. Androgen-Deprivation Therapy and Cardiovascular Disease Risk - The Role of Exercise in Prostate Cancer Treatment. Frontiers in Oncology. 2016;6.

32. Takagi K, Suzuki F, Barrow RE, Wolf SE, Herndon DN. Recombinant human growth hormone modulates Th1 and Th2 cytokine response in burned mice. Ann Surg. 1998;228(1):106-11.

33. Dasu MR, Barrow RE, Spies M, Herndon DN. Matrix metalloproteinase expression in cytokine stimulated human dermal fibroblasts. Burns. 2003;29(6):527-31.

34. Anderson JR. Nerve regrowth and function in burn scars. Perth: University of Western Australia; 2007.

35. Jeschke MG, Gauglitz GG, Kulp GA, Finnerty CC, Williams FN, Kraft R, et al. Long-term persistance of the pathophysiologic response to severe burn injury. 2011.

36. Wall BA, Galvão DA, Fatehee N, Taaffe DR, Spry N, Joseph D, et al. Exercise Improves V˙O2max and Body Composition in Androgen Deprivation Therapy–treated Prostate Cancer Patients. Medicine & Science in Sports & Exercise. 2017;49(8):1503-10.

37. Newton R, Taaffe D, Spry N, Gardiner R, Levin G, Wall B, et al. A phase III clinical trial of exercise modalities on treatment side-effects in men receiving therapy for prostate cancer. BMC Cancer. 2009;9(1):210.

38. Baechle TR, Earle R. Essentials of Strength Training and Conditioning. 3 ed. Champaign, United States: Human Kinetics; 2008.

39. Thompson WR, Gordon NF, Pescatello LS, editors. ACSM's guidelines for exercise testing and prescription. 8th ed: Lippincott Williams & Wilkins; 2010. 72-85.

40. Kenworthy P, Grisbrook TL, Phillips M, Gibson W, Wood FM, Edgar DW. Addressing the Barriers to Bioimpedance Spectroscopy Use in Major Burns: Alternate Electrode Placement. J Burn Care Res. 2017;38(6):e952-e9.

41. Grisbrook TL, Kenworthy P, Phillips M, Wood FM, Edgar DW. Nanocrystalline silver dressings significantly influence bioimpedance spectroscopy measurements of fluid volumes in burns patients. Burns. 2016;42(7):1548-55.

42. Gittings P, Salet M, Burrows S, Ruettermann M, Wood FM, Edgar D. Grip and Muscle Strength Dynamometry Are Reliable and Valid in Patients With Unhealed Minor Burn Wounds. J Burn Care Res. 2016;37(6):388-96.

43. Wu A, Edgar DW, Wood FM. The QuickDASH is an appropriate tool for measuring the quality of recovery after upper limb burn injury. Burns. 2007;33(7):843-9.

44. Gittings PM, Heberlien N, Devenish N, Parker M, Phillips M, Wood FM, et al. The Lower Limb Functional Index - A reliable and valid functional outcome assessment in burns. Burns. 2016;42(6):1233-40.

45. Shaffer S, Harrison A, Brown K, Brennan K. Reliability and validity of semmes-weinstein monofilament testing in older community-dwelling adults. Journal of Geriatric Physical Therapy. 2005;28(3):112-3.

46. Pierce MC, Strasswimmer J, Hyle Park B, Cense B, De Boer JF. Birefringence measurements in human skin using polarization-sensitive optical coherence tomography. Journal of biomedical optics. 2004;9(2):287.

47. Zysk AM, Nguyen FT, Oldenburg AL, Marks DL, Boppart SA. Optical coherence tomography: a review of clinical development from bench to bedside. Journal of biomedical optics. 2007;12(5):051403.

48. Edgar D, Dawson A, Hankey G, Phillips M, Wood F. Demonstration of the validity of the SF-36 for measurement of the temporal recovery of quality of life outcomes in burns survivors. Burns. 2010;36(7):1013-20.
